# A Systematic Review of Expression and Immunogenicity of Human Endogenous Retroviral Proteins in Cancer and Discussion of Therapeutic Approaches

**DOI:** 10.3390/ijms23031330

**Published:** 2022-01-25

**Authors:** Mikkel Dons Müller, Peter Johannes Holst, Karen Nørgaard Nielsen

**Affiliations:** 1 Institute of Immunology and Microbiology, University of Copenhagen, Blegdamsvej 3, 2200 Copenhagen N, Denmark; mikkel@sund.ku.dk; 2 InProTher ApS, Ole Maaløes Vej 3, 2200 Copenhagen N, Denmark; pjh@inprother.com

**Keywords:** human endogenous retroviruses, neoplasms, cancer, cancer treatment, immunogenicity, immunotherapy, tumor-associated antigens

## Abstract

Human endogenous retroviruses (HERVs) are remnants of ancient retroviral infections that have become fixed in the human genome. While HERV genes are typically silenced in healthy somatic cells, there are numerous reports of HERV transcription and translation across a wide spectrum of cancers, while T and B cell responses against HERV proteins have been detected in cancer patients. This review systematically categorizes the published evidence on the expression of and adaptive immune response against specific HERVs in distinct cancer types. A systematic literature search was performed using Medical Search Headings (MeSH) in the PubMed/Medline database. Papers were included if they described the translational activity of HERVs. We present multiple tables that pair the protein expression of specific HERVs and cancer types with information on the quality of the evidence. We find that HERV-K is the most investigated HERV. HERV-W (syncytin-1) is the second-most investigated, while other HERVs have received less attention. From a therapeutic perspective, HERV-K and HERV-E are the only HERVs with experimental demonstration of effective targeted therapies, but unspecific approaches using antiviral and demethylating agents in combination with chemo- and immunotherapies have also been investigated.

## 1. Introduction

Checkpoint inhibitor (CPI) therapy has revolutionized cancer treatment. By circumventing inhibitory immune checkpoints induced by tumor cells, CPIs allow for the (re-)activation of otherwise inactive T cells to directly target the tumor cell and have proven effective in otherwise untreatable cancers [1,2]. However, more than a decade after the approval of the first CPI, ipilimumab, only a subset of patients benefit from CPI therapy [3]. A principal limitation of CPI is that a pre-existing intratumoral immune response is necessary for CPIs to be effective [4]. Prognosis during CPI treatment among different cancers correlates with the tumor mutational burden (TMB) [5] and the presence of cross-presenting dendritic cells [6], corresponding with a prerequisite for cross-presentation of high quality neoantigen epitopes [7]. This trend is consistent across most cancer types, although some types exhibit high inflammation and respond to CPI therapy despite a generally low TMB [8]. This discrepancy has been suggested to be due to the reactivation of human endogenous retroviruses (HERVs) [9,10] that when translated into proteins are indeed antigenic in a tumor context [11]. As HERV genes are mostly silenced in healthy tissues but overexpressed in cancers, they have been proposed to act as a new class of tumor-associated antigens (TAAs) [12] while also functioning as inducers of inflammation through double stranded (ds) RNA [13]. While some recent reviews focused on the latter [14,15], reviews specifically focused on evidence of HERVs as TAAs are limited. 

Eight percent of the human genome consists of HERVs, genetic material that can be traced back to prehistoric retroviral infections [16,17]. HERV genetic fragments display a diversity of structures, ranging from solo long terminal repeats (LTRs) to several partial or intact open reading frames. In specific cases, HERV proteins are even capable of forming particles that can load HERV RNA and reverse transcribe loaded RNA into episomes. However, the last step in the viral replication cycle, integration, appears defective [18]. Consequently, HERV-expressing cells can influence neighboring cells and even in theory cause the expression of new HERV proteins, although actual replication-competent HERVs that can repeat this cycle have not been described [19]. Still, HERV gene products such as group-specific antigen (Gag), envelope (Env), and polymerase (Pol) with reverse transcriptase (RT) activity, Rec and Np9 are occasionally expressed in human cells [20,21,22,23]. Some HERV genes have been exapted into normal cellular functions. In particular, Env of HERV-W and HERV-FRD (also termed syncytin-1 and 2) play a central role in eutherian biology as drivers of proliferation and by mediating membrane fusions for the formation of syncytiotrophoblasts in the placenta [24,25]. Furthermore, HERVs are expressed in pluripotent stem cells [20], where HERV-encoded genes are activated by transcriptional factors related to self-renewal and differentiation [26]. The reverse is also true, as HERV-K is required for neuronal progenitor cell stem cell behavior, effects that in neurons are linked to Env-triggered mTOR signaling pathways [27]. Apart from these defined roles, HERV genes are typically not reported to be transcriptionally or translationally expressed in the healthy, although their expression can be provoked. Upon inflammatory stimuli such as radiotherapy, HERV RNAs are expressed with an excess of antisense RNA, resulting in dsRNA that drives interferon responses; a prime example of how RNA expression data must not be equated to protein translation [28]. Furthermore, HERV proteins have been described to play pathophysiological roles, as is seen in cellular senescence [29], autoimmunity [30,31], neurodegenerative diseases [32,33], and cancer. Protein expression in cancer is a focus point of this review. For detailed descriptions of the pathophysiological processes mediated by HERVs in cancer, we direct the reader to other recent reviews focused on this topic [34,35]. 

Transcription from HERV loci is linked with the demethylation of LTRs, but regulation of the expression is not trivially driven by general demethylation. This is due to retroviral LTRs harboring different transcription factor binding sites and the accessibility of their promoters depending on their genomic location [36,37]. Accordingly, in cancers, transcriptional activation of HERVs can be linked to stemness [38], sex steroid hormones [39], inflammation [40] and potentially to senescent cell phenotypes [41], although the complexity of this mechanism is yet to be fully explored. Furthermore, HERV proteins have been linked to oncogenic signaling pathways [42,43,44], raising the possibility that selective pressure acting upon tumors may lead to increased HERV expression. As an example, in otherwise superficially similar basal-type triple-negative breast cancer sequenced for the expression of four HERV-K loci, tumors were found to either have high HERV-K expression or *KRAS* mutations, but not both [45].

With such pleiotropic functions in tumors and their abundant presence in the genome, several strategies for HERV specific medical use have been proposed. These include: (1) the use of HERV RNA, protein or immunity as biomarkers [46]; (2) blocking the transcription of HERVs in tumor cells using small interfering (si) RNA or CRISPR [47]; (3) demethylating agents that increase expression of HERV RNA, leading to the generation of dsRNA and interferons [48,49]; (4) blocking the function of HERV proteins with antiviral drugs [50]; and (5) targeting HERV-specific antigens with immune stimulation to mount an adaptive immune response towards HERV-expressing cells [51].

However, no clinically approved diagnostic tests detecting HERVs or therapies specifically targeting HERVs for the treatment of cancer exist today. In order to support such efforts, this review systematically investigates the relationship between specific HERV proteins and cancer types. Several reviews describe this subject, but to our knowledge, only one of these is systematic, and its scope is limited to specific surgically relevant diseases [52]. No reviews have to date focused on the crucial distinction between RNA and protein expression in human cancers. RNA expression can either be interpreted as a proxy for protein expression or as an independent inducer of the interferon response through dsRNA. This distinction is important as immune mechanisms exist for HERV RNA expression without translation into protein, and as protein expression is critical if HERVs are to function as direct targets of immunotherapy. Here, we provide an updated, systematic review of studies that evaluate HERV protein expression as well as adaptive immune responses towards HERVs in cancer and tumor cell lines. We include all neoplasms and also describe in vitro and in vivo studies where relevant. It is our hope that this will provide an overview in the field of HERVs in cancer and illuminate potential strategies on how HERV proteins may be further explored and hopefully exploited therapeutically as targets of immunotherapy.

## 2. Materials and Methods

### 2.1. Information Sources

The literature search was conducted using the Medical Search Headings (MeSH) function in pubmed.ncbi.nlm.nhs.gov (accessed on 2 November 2021). A search was performed using the search terms “Endogenous Retroviruses” [Mesh] AND “Neoplasms” [Mesh]. All hits were allocated to either of two categories: “individual papers” and “reviews”, according to whether the paper contained original data. All references from relevant reviews were screened for eligibility by means of the evaluation of the publication titles. Eligible papers were added to the papers identified in the initial search. All individual papers were then evaluated fully in accordance with the eligibility criteria described below. Included papers were then stratified in accordance with the data items listed below. Additional papers were identified using complementary nomenclature based on appearance in references in the original dataset or when searched for during the discussion of the primary dataset.

### 2.2. Eligibility Criteria and Data Management

Eligibility criteria: Papers were selected based on whether or not they concerned neoplasms and HERVs. Therefore, papers concerning other diseases and/or other species of retrovirus were excluded. In addition, papers were only selected if they showed evidence of HERV translational activity. This was defined as immunoblot assays, flow cytometry, and immunohistochemical assays. Indirect assays such as measurement of immune reactivity by antibody ELISA and specific T cell assays were also included and taken as evidence of antigen encounter. Papers providing only transcriptional evidence were excluded. Finally, papers were only included if they were accessible and available in English. 

Data management: Each paper was tracked using unique PMID or DOI and data extraction was performed with Microsoft Excel. 

Data items: Identified papers were stratified into neoplasm type and HERV type in addition to result, broadly entered into the following categories: expression (defined as direct evidence of protein expression), T cell activity (defined as T cell presence, activation or reactivity), seroreactivity (defined as assays detecting serum antibodies), therapeutics (defined as a study investigating a potential therapy in vitro or in vivo), tumor behavior (defined as assays designed to investigate HERV roles in tumors) and prognostic/diagnostic (defined as papers investigating possible diagnostic and or therapeutic aspects of HERVs in cancer). While naming guidelines do exist [53,54], we generally adhered to the naming used in the identified publications. Additional information on study design is shown in tables including tissue type investigated, cell lines used, type of controls used and number of cases and controls. Controls were broadly categorized as adjacent (defined as using tissue adjacent to lesion) and healthy controls (defined as tissue or blood sample from a person without cancer). Finally, some papers were given more specific categories, e.g., “Blood from KSHV-negative patients”, to better condense the study design. Assessment of data items was conducted once for excluded papers and twice for included papers.

### 2.3. Search Results

**Figure 1:**
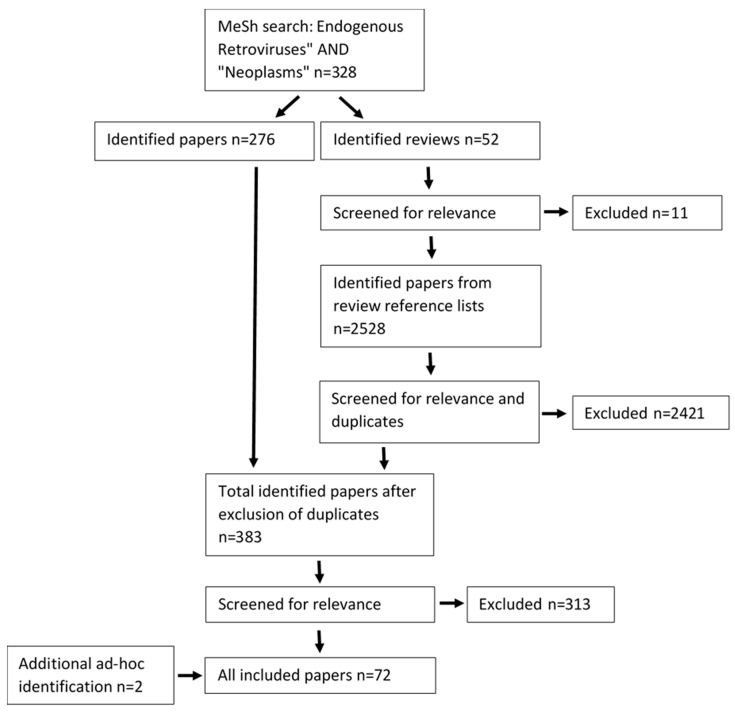
Flowchart on how papers were found and evaluated.

The initial search was performed as described above and as visualized in Figure 1 on 2nd November 2021 and yielded 328 results. Fifty-two reviews were identified, of which 11 were inaccessible, not written in English or irrelevant to either HERVs or cancer and were excluded. The remaining 41 reviews contained 2528 references that were screened for relevance in titles, excluding duplicates of papers and papers not concerning HERVs or cancer. A total of 107 papers were identified this way. Finally, 383 original papers were identified, 313 of which were excluded because they were inaccessible, not written in English, only provided transcriptional evidence of HERV expression or for being irrelevant to HERVs or cancer. Seventy papers were included in the tables of this review. An additional 2 papers were added as described above in Section 2.1. The total number of papers included was 72.

## 3. Results and Discussion

### 3.1. Germ Cell Cancers

Germ cell tumors (GCT) have been known to express HERV proteins for decades [55]. Therefore, this category includes a number of older publications (Table 1) that primarily used the detection of serum antibodies as a surrogate to detect HERV protein expression [55,56,57,58,59,60]. 

Loss of methylation increases HERV-K Gag protein expression in teratocarcinoma cell lines [61]. Additionally, HERV-K protein expression is shown to interfere with processes during spermatogenesis also thought to be involved in tumor development [62]. In accordance with studies in other cancers, one publication further found that HERV-K is potentially involved in oncogenesis, promoting migratory properties as well as resistance to chemotherapy [63]. GCTs were reported to express HERV-K proteins capable of forming retroviral particles containing retroviral RNA through multiple protein expression patterns [64,65].

Humoral responses toward HERV-K have been detected in patients with a wide range of GCTs with a clear correlation between increased titers and poor outcome [66]. Cellular responses toward HERV-K Gag were found in seminoma patients [67].

**Table 1:** HERV protein expression in germ cell cancers.

Indication	HERV	Tissue	Cell Line	Controls	Case/Control	Conclusion	Reference
Teratocarcinoma	HERV-K	-	NCCIT	-	-	Expression Tumor function	[63]
Teratocarcinoma	HERV-K	-	Tera-1	-	-	Expression Tumor function	[64]
Teratocarcinoma	HERV-K	-	Tera-1, Tera-2, NCCIT, 2102Ep, PA-1	-	-	Expression	[65]
Teratocarcinoma	HERV-K	Serum	GH	-	Unclear	Expression Seroreactivity	[59]
Teratocarcinoma	HERV-K	-	GH, Tera-2, MRC-5	-	-	Expression	[58]
Teratocarcinoma	HERV-K	-	Tera-1, Tera-2	-	-	Expression	[56]
Teratocarcinoma	HERV-K	Tumor	Tera-1, PA-1	-	8/0	Expression Tumor function	[61]
Testicular cancer	HERV-K	PBMCs	-	Healthy controls	26/18	T cell activity	[67]
Testicular cancer	HERV-K	Serum Tumor	Tera-1	-	57/0, 8/0	Seroreactivity	[55]
Multiple GCT	HERV-K	Serum	-	Serum	Unclear	Seroreactivity Prognostic	[66]
Multiple GCT	HERV-K	Serum	-	-	52/84	Seroreactivity	[60]
Multiple GCT	HERV-K	Serum	-	-	49/15	Seroreactivity	[57]
Unspecified GCT	HERV-K	Serum	-	-	18/0	Tumor function Seroreactivity	[62]

### 3.2. Neurological Cancers

For decades, only a few studies reported translational data in neurological cancers (Table 2) with reports of seroreactivity in brain tumor patients [55] and Np9 expression in the U87 glioblastoma cell line [43]. This changed in 2021 when Doucet-O’Hare et al. observed that *SMARCB1* deletion in atypical teratoid rhabdoid tumors (AT/RT) results in C-MYC translocation to HERV-K 5′ LTRs, leading to HERV-K transcription, translation, and secretion of the Env protein in extracellular vesicles. Notably, targeting HERV-K with CRISPR-dCas9 leads to cancer cell death, and partial interference with siRNA and short hairpin (sh) RNA decreases Ras expression [68]. A recent paper on Moezin-ezrin-radixin-like protein (MERLIN) deficient gliomas and schwannomas by Maze et al. was separately identified. Here, the authors describe how the tumors strongly overexpress HERV-K proteins, including Env at the cell surface and on exosomes, and that such tumors are sensitive to antiretroviral drugs and HERV-K Env-specific monoclonal antibody treatment [69].

### 3.3. Skin Cancers

HERV-K protein expression has been reported in dermatological cancers since the early 2000s [70,71,72], in particular in melanoma (Table 3). One study found that stress induced by serum starvation provokes a non-adherent more malignant phenotype of melanoma cells which is accompanied by an increase in HERV-K protein expression and the release of virus-like-particles. Furthermore, this phenotype showed a reduction in differentiation antigen melan-A/MART-1 as well as human leucocyte antigen (HLA) class I [73]. Additionally, HERV-K was found to play a significant role in proliferation of the A-375 melanoma cell line in vivo and in vitro [74]. 

HERV-K-specific antibodies are found in the serum of melanoma patients in contrast to serum from healthy controls, suggesting that adaptive immune responses toward HERV-K occur naturally and that HERV-K is a viable B cell immunogen [75]. Additionally, increased HERV-K antibody titers correlate with poor prognosis and are increased when melanomas originated in sites not exposed to sunlight [76].

One study investigated HERV-K Env through immunohistochemistry as part of a target validation and found that 30% of primary melanoma samples stained positive, while 29 different healthy tissues from three organ donors were negative. Building on this, the authors engineered HERV-K-specific chimeric antigen receptor (CAR) T cells that significantly reduced tumor burden in a xenogeneic mouse model, providing suggestive evidence that using HERV-K as a T cell target may be feasible [77]. An interesting but less specific therapeutic approach was found in a nude mouse model where growth of a HERV-K expressing human melanoma xenograft was reduced using the reverse transcriptase inhibitor (efavirenz) [78].

HERV-W is studied less often and has only been reported in cutaneous T cell lymphoma (CTCL) [79], where HERV-W Env was found to promote fusogenic properties in CTCL cell lines, while significantly less HERV-W is translationally expressed in non-transformed T cell lines [80]. This is in accordance with reported functions of HERV-W proteins in placental cells [24,25]. Notably, this role in promoting cell fusion is also reported in melanomas expressing HERV-K [42].

Lastly, HERV-H is found in melanoma cell line Hs294T, where H17 peptide is associated with the oncogenic properties de-differentiation and immune escape [81].

**Table 3:** HERV protein expression in skin cancers.

Indication	HERV	Tissue	Cell Line	Controls	Case/Control	Conclusion	Reference
CTCL	HERV-W	-	Mac-1, Mac-2A, MyLa	-	-	Expression Tumor function	[80]
CTCL	HERV-W	Tumor		Non-malignant skin lesions	26/5	Expression	[79]
Melanoma	HERV-H	-	Hs294T	-	-	Expression Tumor function	[81]
Melanoma	HERV-K	Tumor	A-375-SM	Healthy controls	220/55	Expression Therapeutic	[77]
Melanoma	HERV-K	Tumor	-	Naevi	35/38	Expression Tumor function	[42]
Melanoma	HERV-K	-	TVM-A12, TVM-A197	-	-	Expression Tumor function	[73]
Melanoma	HERV-K	Serum	-	Healthy controls	312/70	Expression Prognostic	[76]
Melanoma	HERV-K	-	A-375	-	-	Expression Tumor function	[74]
Melanoma	HERV-K	-	SK-MEL-28	-	-	Expression	[72]
Melanoma	HERV-K	Tumor	SK-MEL-28	-	53/0	Expression	[71]
Melanoma	HERV-K	Serum	-	Healthy controls	81/95	Seroreactivity	[75]
Melanoma	HERV-K	-	A-375	-	-	Expression Tumor function Therapeutic	[78]
Melanoma	HERV-K	PBMCs	-	-	1/0	T cell activity	[82]
Melanoma	HERV-K	Primary tumor and metastases	SK-MEL-28, SK-MEL-1	Naevi	Unclear	Expression	[70]

CTCL: cutaneous T cell lymphoma.

### 3.4. Hematological Cancers

Evidence of HERV translation exists in both myeloid and lymphoid leukemias (Table 4). Although HERV-K and HERV-W have been studied most extensively, a recent paper reported T cell reactivity towards a wide range of HERVs in patients with myeloid malignancies [83].

HERV-K Gag and Env were reported to be processed and expressed in both lymphoma and thrombocytopenia patients, accompanied by evidence of subsequent protein packaging into retroviral particles [21,84,85].

The published literature describes both upstream regulation of HERV expression as well as downstream effects of increased HERV activity [43,86]. Alqahtani et al. found that cellular stress from contact with silver nanoparticles increases HERV-W Env protein expression through a possible survival response to an increase in reactive oxygen species (ROS) [86]. Huang et al. found that HERV-K encoded Np9 co-activates Wnt/β-catenin, Notch1, ERK, and Akt signaling pathways, leading to increased survival and proliferation of leukemia cells [43]. Taken together, a pattern emerges of stress leading to HERV protein expression, which leads to cancer cell survival and proliferative abilities.

Saini et al. found that T cells specific for a wide range of HERVs are present in patients with myeloid malignancies at higher levels than in healthy donors [83]. This study furthermore confirmed HLA class I presentation of HERV peptides, underscoring the potential for therapies that induce HERV-targeting T cells. Notably, after treatment with the demethylating agent 5-azacytidine, known to upregulate HERV transcription, the authors did not find systematic differences in HERV-specific T cell responses. Thus, whether demethylating agents regulate HERV protein remains to be established [83].

With regard to cancer diagnosis, mRNA expression of HERV-W Env/syncytin-1 in the blood of leukemia patients is accompanied by anti-syncytin-1 binding antibodies. Furthermore, HERV-W Env expression was observed in 43 out of 57 patients and none of the 20 healthy controls, making HERV-W a potential diagnostic marker in leukemia [87]. This suggests that HERV-W is immunogenic and that a response towards it is natural and does not pose immediate safety concerns. Similar seroreactivity toward HERV-K Gag was seen in patients with lymphomas [55].

**Table 4:** HERV protein expression in hematological cancers.

Indication	HERV	Tissue	Cell Line	Controls	Case/Control	Conclusion	Reference
AML, MDS, CMML	HERV-K HERV-H HERV-W HERV-FRD HERV-E	PBMCs Bone marrow	-	Healthy controls	22/27	T cell activity	[83]
AML, ALL	HERV-W	-	FA-AML1, MOLT-4	-	-	Expression Tumor function	[86]
AML, CML, ALL Multiple myeloma	HERV-K	Blood	K562, K562/adr, KCL-22, KCL-22M, KG-1, HL-60, NB4, Kasumi-1, Jurkat, Molt-4, H9, Raji, KM3, 8226	Healthy controls	50/22	Expression Tumor function	[43]
CLL, CML, ALL, AML, AMLL, NHL	HERV-W	Blood	-	-	57/20	Expression Prognostic	[87]
B cell lymphoma	HERV-K	-	JVM2, REC1	-	-	Expression	[85]
Large cell lymphoma, mantle cell lymphoma	HERV-K	Blood		Healthy controls	3/3	Expression	[84]
CML, ET	HERV-K	Blood	-	Healthy controls	2/2	Expression	[21]
ALL and lymphomas	HERV-K	Serum	-	-	81/0 227/0	Seroreactivity	[55]

ALL: acute lymphoblastic leukemia. AML: acute myeloid leukemia. AMLL: acute mixed lineage leukemia. CLL: chronic lymphoblastic leukemia. CML: chronic myeloid leukemia. CMML: chronic mixed myeloid leukemia. ET: essential thrombopenia. MDS: myelodysplastic syndrome. NHL: non-Hodgkin’s lymphoma.

### 3.5. Prostate Cancer

HERV-K is the only HERV that has been studied at the protein level in prostate cancer. In comparison to other cancer types, these studies include large patient cohorts, and several therapeutic, diagnostic, and prognostic opportunities are suggested (Table 5).

As in other cancer fields, HERV-K protein expression is increased in malignant regions of tissue samples as compared to adjacent benign regions [88]. One study found that PC-3 xenografted nude mice have reduced tumor burden when both pre-treated and treated with the antiretroviral compound efavirenz [78]. This may suggest functional retroviral reverse transcriptase in this cell line, although the effect of efavirenz may also be exerted on other retroelements [89]. Targeting HERV-K Env by CRISPR/Cas9 significantly downregulates prostate cancer cell line expression of proto-oncogene SF2/ASF as well as the RAS pathway [90].

Notably, two publications included large patient cohorts with translational data [46,91]. Wallace et al. included 377 prostate adenocarcinoma tissue samples for immunohistochemical staining along with control tissue of benign prostate hyperplasia. Furthermore, 294 peripheral blood mononuclear cell (PBMC) samples along with 135 PBMC samples from healthy controls were evaluated for HERV-K RNA. Elevated HERV-K Env protein was correlated with disease, age, and smoking status. Additionally, increased HERV-K *env* and *gag* mRNA in blood samples correlated to prostate cancer diagnosis, suggesting that it may be used as a diagnostic tool along with prostate-specific antigen (PSA) [46]. Reis et al. included 188 prostate cancers and 22 controls for immunohistochemical staining, finding that upwards of 85% of samples stained positive for HERV-K Gag. Moreover, analysis of serum from 483 prostate cancer patients and 148 healthy donors for HERV-K antibody detection by ELISA showed a correlation between HERV-K Gag antibodies and prostate cancer stage. Finally, 284 patients were included in a follow-up analysis that showed worse prognosis when anti HERV-K Gag antibodies were detected [91]. Combined with other autoantibodies, these provide a diagnostic marker that was shown to distinguish prostate cancer patients from healthy controls [92]. Overall, the reported protein expression data confirm a highly consistent association between primary prostate cancers and HERV-K. This is in agreement with otherwise reported highly prominent transcriptional expression of HERV-K from an androgen-dependent locus with a number of other loci also detected by the sequencing of PCR products [93].

**Table 5:** HERV protein expression in prostate cancers.

Indication	HERV	Tissue	Cell Line	Controls	Case/Control	Conclusion	Reference
Adenocarcinoma	HERV-K	-	LNCaP	-	-	Expression Tumor function Therapeutics	[90]
Adenocarcinoma	HERV-K	Tumor Blood	CWR22, 22Rv1, PC-3 and DU145	Healthy controls	377/0 294/135	Expression Diagnostic	[46]
Adenocarcinoma	HERV-K	-	PC-3	-	-	Expression Tumor function Therapeutic	[78]
Prostate cancer	HERV-K	Tumor	-	Adjacent	18/18	Expression Tumor function	[88]
Prostate cancer	HERV-K	Serum	-	Serum	Unclear	Expression Diagnostic	[92]
Prostate cancer	HERV-K	Tumor Serum	Vcap, LNcap, PC-3	Healthy controls	188/22 483/148 284 *	Tumor function Seroreactivity Prognostic	[91]

* Two hundred and eighty-four of the 483 patients were included in a follow up.

### 3.6. Mammary Cancer

Only HERV-K is found in mammary cancers (Table 6), and the majority of the identified studies were performed by Wang-Johanning and associates [94,95,96,97,98], who also performed studies of HERV-K in ovarian and pancreatic cancers described later [99,100,101]. 

There is evidence that HERV-K protein expression in breast cancer regulates epithelial to mesenchymal transition (EMT) as well as genes associated with invasion and metastases [98]. Additional evidence of HERV-K correlating with metastases can be seen in a study using an in vivo mouse model that shows an increase in lymph node metastases in mice xenografted with HERV-K expressing tumors, compared to tumors not expressing HERV-K [95].

Both humoral and cellular HERV-K-specific responses are reported in breast cancer patients with patient PBMCs showing lysis specifically in HERV-K expressing breast cancer cells [96]. Expanding on this, a mouse model with xenografted HERV-K expressing tumors was successfully targeted with HERV-K Env directed monoclonal antibody treatment [95].

The impact of HERVs being exploited in diagnostics or prognostics has also been investigated [22,94,96,97]. Wang-Johanning et al. found increased levels of HERV-K Env directed antibodies in cancer patients [94]. Coupled with serum HERV-K RNA this provides diagnostic potential when compared with healthy controls [96]. Furthermore, increased HERV-K reverse transcriptase expression significantly correlates with poor prognosis [22]. This was similarly reported for HERV-K Env protein, where high expression in tumor biopsy sections correlated with worse patient outcome [97].

**Table 6:** HERV protein expression in mammary cancers.

Indication	HERV	Tissue	Cell Line	Controls	Case/Control	Conclusion	Reference
DCIS and IDC	HERV-K	Tumor	MDA-MB-231, Hs578T, MCF-7, SKBR3 and 293TN	-	-	Expression Tumor function	[98]
DCIS and IDC	HERV-K	Serum	-	Healthy controls	49/13	Seroreactivity Diagnostic	[96]
DCIS and IDC	HERV-K	Tumor	MDA-MB-231, SKBR3, MDA-MB-453, T47D, and ZR-75-1	Adjacent	2/2	Expression Therapeutic	[95]
DCIS and IDC	HERV-K	Tumor	-	Adjacent Healthy controls	110/30 195/0	Expression Prognostic	[97]
DCIS, IDC, IMPC, SCC	HERV-K	Tumor		Healthy controls	119/63	Expression T cell activity	[94]
Breast cancer	HERV-K	Tumor		Adjacent	110/85	Expression Prognostic	[22]
Adenocarcinoma	HERV-K	-	T47D	-	-	Expression Tumor function	[102]
Breast cancer	HERV-K	Serum	-	-	103/0	Seroreactivity	[55]

DCIS: ductal carcinoma in situ. IDC: invasive ductal carcinoma. IMPC: invasive micropapillary carcinoma. SCC: squamous cell carcinoma.

### 3.7. Gastrointestinal Cancers

Only publications concerning colonic and rectal cancers were identified in our search (Table 7). Among these, translation of HERV-K, HERV-H, HERV-W, HERV-FRD and HERV-3 was studied. 

HERV-K is found to be instrumental in cancer cell colonization, proliferation, and migration in vitro, and this translates to increased tumor growth in an in vivo mouse model. The findings of this study suggest that this is closely related to the *NUPR1* gene and ROS [44]. Furthermore, the immunosuppressive domain (ISD) of the Env protein has been hypothesized to contribute to immune evasion, as was confirmed by administering HERV-H derived ISD peptide and by knockdown of HERV-H *env*. Here, HERV-H was shown to be involved in the upregulation of CCL19, resulting in the recruitment of immunoregulatory CD271+ cells through the Twist-PI3K pathway [81]. Ferrari et al. further categorized exosomes from cancer cell lines using antibodies toward HERV-K and HERV-W Env in flow cytometry, suggesting intercellular signaling effects of HERV containing exosomes such as immune regulation [103]. Both Ferrari et al. and Mullins et al. addressed the scarcity of specific antibodies as a recurring issue in procuring translational data in the HERV space [103,104].

An antiviral therapy approach was tested in this cancer field by Díaz-Carballo et al., who found that increased chemotherapy resistance in cancer cells correlates with an increase in HERV expression, and that administration of antiviral drugs amantadine, ribavirin and pleconaril can mitigate this resistance [50]. This approach provides insights into possible therapeutic strategies as well as HERV significance in cancer disease. This is in accordance with a study in lung cancer described below [78].

Of prognostic relevance, HERV-W expression is a marker of poor prognosis in rectal but not in colonic cancer [105]. 

**Table 7:** HERV protein expression in gastrointestinal cancers.

Indication	HERV	Tissue	Cell Line	Controls	Case/Control	Conclusion	Reference
Colorectal cancer	HERV-K	-	DLD-1 and HCT116	-	-	Expression Tumor function	[44]
Colorectal cancer	HERV-K HERV-W	-	Caco-2, SK-CO-1	-	-	Expression Tumor function	[103]
Colorectal cancer	HERV-H	Tumor	-	-	13/13	Expression	[104]
Colorectal cancer	HERV-W HERV-FRD HERV-3	Tumor	HCT8	-	20/0	Expression Tumor function Therapeutic	[50]
Colorectal cancer	HERV-H	-	Colo320 and HCT116	-	-	Expression Tumor function	[81]
Colorectal cancer	HERV-W	Tumor	-	-	140/0	Expression Prognostic	[105]
Colorectal cancer	HERV-K	-	HT29	-	-	Expression Tumor function Therapeutic	[78]

### 3.8. Gynecological Cancers

Evidence of HERV protein expression has frequently been observed in gynecological cancers, and a broad spectrum of HERVs have been reported (Table 8). HERV-W is widely reported across these indications [106,107,108]. Ovarian cancer is the only tumor type in which we identified expression of human endogenous MER34 ORF (HEMO) Env [109].

Several tumor functions have been reported to be affected by HERVs [110,111,112]. HERV expression is closely related to methylation levels in endometrial carcinoma, and a lower state of differentiation is correlated with increased HERV expression [112]. Though some regulatory factors are reported, the exact mechanisms are not fully understood [113].

HERV-K has been tested as a potential target for adaptive cellular immune responses in both ovarian cancer and choriocarcinoma [100,110]. Rycaj et al. further showed that HERV-K-specific T cells expanded from ovarian cancer patients are enriched in ascites fluid as compared to peripheral blood [99]. Using a different approach, a hallmark paper by Chiappinelli et al. found that demethylation treatment of ovarian cancer cells leads to the upregulation of HERV dsRNA, ultimately resulting in an increase in innate immune activity [48]. A similar approach with the potential to act via the induction of HERVs and antiviral mimicry was performed by Díaz-Carballo et al. by xenografting mice with the SKOV3 ovarian cancer cells and showing decreased tumor burden by combination therapy with the demethylating agent romidepsin and the antiviral vesatolimod [106].

**Table 8:** HERV protein expression in gynecological cancers.

Indication	HERV	Tissue	Cell Line	Controls	Case/Control	Conclusion	Reference
Endometrial carcinoma	HERV-W HERV-FRD ERV-3	Tumor	RL95-2	7 adjacent/22 age matched	38/29	Expression Tumor function	[112]
Endometrial carcinoma	HERV-W	Tumor	-	12 adjacent/12 age matched	24/24	Expression	[107]
Ovarian	HERV-W HERV-FRD HERV-V ERV-3	Tumor	SKOV3 OVCAR	-	10/0	Expression Tumor function Therapeutic	[106]
Ovarian	HERV-K ERV-3	Tumor	-	Healthy controls	70/10	Expression Tumor function	[113]
Ovarian	HEMO	Serum Tumor	HeLa	-	Unclear	Expression Tumor function	[109]
Ovarian	HERV-K	Tumor cysts	-	Adjacent	89/89	T cell activity eroreactivity	[99]
Ovarian	HERV-W ERV-3	-	A2780	-	-	Expression Tumor function Therapeutic	[48]
Ovarian	HERV-K ERV-3	Tumor Serum	SKOV3, OVCA 430, OVCA 433, OVCA 420, OVCAR3, DOV 13 and OVCA 429, T29, T72 and T80	Healthy controls	553/3 20/20	Seroreactivity	[100]
Choriocarcinoma	HERV-W HERV-FRD HERV-K	-	BeWo, JEG	-	-	T cell activity	[110]
Choriocarcinoma	HERV-W	-	JAR, JEG-3	Normal trophoblast	-	Expression	[108]
Choriocarcinoma	HERV-E	-	JEG-3, JAR, BeWo, HeLa	Normal trophoblast		Expression	[111]

### 3.9. Urological Cancers

Although clear cell renal cell carcinomas were mainly found to have been studied (Table 9), one study investigating urothelial cell carcinoma (UCC) was identified [114]. Here HERV-W was shown to be upregulated by oncogene C-MYB and promote growth, while also correlating with cancer stage. Crucially, a large number of cases were linked to mutations in the syncytin-1 3′-UTR and the mutated sequence had increased binding by C-MYB [114]. Confirming this finding in independent studies would provide proof of a causal involvement in tumor generation and/or progression. 

For clear cell renal cell carcinoma (ccRCC), a cancer that has a high degree of inflammation despite a low TMB, a larger number of HERVs are believed to be reactivated at the RNA level, contributing to inflammation [10], but only HERV-E and HERV-K are published at the protein level (Table 9). T cells targeting HERV-E were identified in patients responding to hematopoietic stem cell transplantation. Additionally, transcripts of HERV-E were detected in ccRCC biopsies and cell lines, but not in other cancers and healthy tissues [115]. This is subsequently backed up by evidence of protein expression by the same group [116] who further found HERV-E to be regulated by the tumor suppressor gene von Hippel-Lindau (VHL), which has a strong association with ccRCC. The specific HERV-E epitopes were later identified [117], and currently a clinical trial is ongoing with autologous T cell therapy specifically targeting one of these peptides (NCT03354390 [118]).

HERV-K Env protein expression was recently also detected in ccRCC in a large cohort. Survival data from this study indicate that the subcellular localization of the Env protein may serve as a prognostic marker as an increase in tumor grade, correlating with lower membranous expression and higher cytosolic expression of HERV-K [119].

**Table 9:** HERV protein expression in urological cancers.

Indication	HERV	Tissue	Cell Line	Controls	Case/Control	Conclusion	Reference
Urothelial cell carcinoma	HERV-W	Tumor	-	Adjacent	82/82	Expression Tumor function Prognostic	[114]
ccRCC	HERV-K	Tumor	MZ1257RC	-	288/0	Expression Tumor function Prognostic	[119]
ccRCC	HERV-E	Tumoral T cells	-	Healthy controls	4/4	T cell activity	[10]
ccRCC	HERV-E	PBMCs	-	-	7/0	T cell activity Therapeutic	[117]
ccRCC	HERV-E	Tumor	-	-	67/17	Expression Tumor function	[116]
ccRCC	HERV-E	PBMCs	Multiple RCC cell lines	-	1	T cell activity	[115]

ccRCC: clear cell renal cell carcinoma. PBMCs: peripheral blood mononuclear cells.

### 3.10. Lung Cancers

Few publications with translational data of HERV-K were found in the field of lung cancers (Table 10). The identified studies did not focus mainly on lung cancers and they did not include patient samples [43,78]. One study found that mice xenografted with the cell line H69 had a reduced tumor burden when treated or even pre-treated with antiretroviral compound efavirenz [78], as was also found in a colorectal cancer model [106].

### 3.11. Pancreatic Cancers

HERVs were not found to be extensively studied in pancreatic cancers (Table 11). Kudo-Saito et al. described the expression of HERV-H Env in a pancreatic cell line and observed reduced immunogenicity following HERV-H knockdown [81]. Chen et al. confirmed protein expression of HERV-K Np9 on the same cell line [43]. Li et al. performed a dedicated target validation study and initially focused on characterizing libraries of pancreatic cancer tissues. Here they found prominent HERV-K expression [101]. The authors further found that downregulation of HERV-K expression in pancreatic adenocarcinoma cells impairs tumor functions through RAS-ERK-RSK pathways in several cell lines and observed reduced tumor formation in mouse xenograft models treated with HERV-K Env targeting shRNA [101].

### 3.12. Endocrine Cancers

Only one study was identified, providing evidence of HERV-W protein expression in this category (Table 12). This publication addressed insights into HERV-W function in pituitary adenomas where gene expression closely correlates to cAMP pathways, and observed upregulation in adenomas as compared to non-neoplastic tissue [120].

### 3.13. Sarcomas

Only one publication found HERV-K protein in HIV-associated Kaposi’s sarcoma (Table 13). HERV-K transcriptional levels were found to correlate with transactivation with Kaposi’s sarcoma associated herpes virus and this was involved in cell invasiveness, possibly through Np9 [121].

### 3.14. Discussion

#### 3.14.1. Underexplored HERVs in the Published Literature

Our analysis reveals that most published studies identified a positive correlation between HERV protein expression and cancer. This may at least partly rely on publication bias, with negative correlations less likely to be published. However, the studies that include a non-cancerous control—be it cell line or healthy tissue—found that HERVs have significantly higher protein expression levels in neoplastic samples. The volume of publications included in this systematic review was strongly limited by the availability of data on HERV translation. The majority of initially identified publications detecting tumoral HERV expression were excluded as they investigated HERV only at the RNA level. This is a major limitation in the available data. As Ferrari et al. and Mullins et al. addressed in their discussions, there is an eminent bias with procuring translational data, namely the availability of HERV-specific antibodies [104,119]. 

Addressing HERV expression from RNA sequencing data could be an alternative, although this approach also has several limitations. Due to their hundreds of highly homologous LTR sequences and the presence of splice variants, HERVs are inherently difficult to identify from sequencing data, and are often either discarded in data cleaning or poorly annotated. Thus, the published literature may not necessarily reflect the HERV transcriptional landscape. Likewise, it is important to factor in that transcription does not necessarily result in translation, and since HERV dsRNA has immunomodulatory capacity [48,49], parsing out the contributions from the innate immune response and the adaptive response towards HERV-derived epitopes may be difficult. A broader approach that allows for a comparatively unbiased identification of protein expression is LC/MS, as has been applied in HERV transfected cells [122]. Using LC/MS global protein expression analysis is possible, but it can be difficult if the protein sequences are poorly annotated and if the expression levels are insufficient.

As HERV-K, HERV-W, HERV-H are the HERVs that have received by far the most attention in the scientific community, it is to be expected that these will also be most widely represented in our analysis. We do find HERV-K (detected in 12 of the 13 cancer types) and to a lesser extent HERV-W (detected in 7 of the 13 cancer types) to dominate the published literature, with HERV-H being considerably less prevalent. What underlies this discrepancy is unknown. In this context, it is particularly interesting that Saini et al. identified an HERV-H epitope to be the most immunogenic in myeloid malignancy patients when compared with several HERVs, even though HERV-H translation has not yet been detected in hematological cancers. Of note, even the selection of HERV epitopes in the Saini et al. study was not unbiased, as the authors focused solely on HERVs that were systematically annotated [83].

#### 3.14.2. Historical Context

A clear trend emerges of some cancer fields having been investigated more and with varying degrees of methodological sophistication. Historically, HERVs were studied in GCTs such as seminomas throughout the 1990s, whereas HERVs were curiously thoroughly studied as potential targets of immunotherapy in melanoma by numerous groups in the mid-2000s, before being somewhat neglected by the field (Table 3). This suggests that contemporary attention is a determinant of how frequently HERVs have been studied. One could speculate that attention shifted as melanomas subsequently received intense attention as prominent responders to checkpoint blockade therapy, with the response rate being largely driven by T cells recognizing somatic mutations [8]. In contrast, in the field of mammary and ovarian cancer, Wang-Johanning and associates drove the production of studies, showcasing that the attention of specific research groups may also affect how and what fields are studied. 

Different cancer fields have entered the HERV space from somewhat different angles. Colorectal and hematological cancer research concern tumor functions and immune activity to a greater extent (Table 4 and Table 7). Prostate cancers are studied with large patient sample sizes in regard to using HERVs in cancer diagnosis and prognosis (Table 5). 

Head and neck cancers did not produce any hits in the search for this review, which may indicate that HERVs are not expressed in these cancers. Evidence however suggests that HERV gene products are indeed present. In 1987, Tan et al. showed that an antibody specific for the murine γ-retrovirus p15E ISD binds head and neck cancers [123]. Even though the antibody target was speculated to be of endogenous origin, the source of protein expression was not determined. Serum from head and neck cancer patients exhibited an immune-suppressive function in macrophage polarization assays, and this ability could be blocked by antibodies specific for murine γ-retrovirus p15E immunosuppressive domain [123,124]. A much more recent study confirmed that HERV-H, also of the γ-retrovirus family, is differentially transcribed in head and neck cancers as the only HERV consistently identified [125], indicating that this may be the factor identified by Tan et al. Likewise, HERV-K RNA has been detected in hepatocellular carcinoma [126] and HERV-K and W RNA in neuroblastoma cell lines [127,128]. Thus, there is still exciting ground to cover in the field of HERVs and cancer, and studies systematically evaluating HERV translation in cancer may pave the way for new therapeutic targets and modalities.

#### 3.14.3. Biological Function in Cancer Development and Progression

While a causal role for HERVs in directing the development of human cancers is not proven, some trends are apparent throughout this analysis. HERVs seem to be consistently correlated with EMT, where HERV-K Np9 and Env play putative roles [43,63]. Furthermore, a number of studies targeted HERV-K with CRISPR/Cas9, RNAi, shRNA or monoclonal antibodies, and uniformly found diminished migratory, proliferative and/or survival capacity of tumor cells expressing HERV-K protein [44,68,90,95,96,98,101]. These studies are supported by Lemaître et al., who observed corresponding findings by HERV-K transcriptional and translational overexpression [129]. Furthermore, the genetic link between *MERLIN* or *SMARCB1* deficiency leading to HERV-K overexpression and cancers that depend on HERV-K for survival is quite strong [68,130], indicating that HERV-K may play a functional role in cancer development and progression. 

While not nearly as frequently reported with protein expression as HERV-K in cancer, HERV-W/syncytin-1 presents the possibly strongest suggestion of a causal relation, as studies of bladder cancers identified a recurrent somatic mutation in the 3′-UTR of the syncytin-1 gene, which increases its protein expression [114]. If such mutations were to be seen in other studies, we would have confirmation that HERV expression is selected for during tumor progression as opposed to a consequence of cancer progression.

As with HERV-W, we found limited functional studies on HERV-H protein expression. The reports had different read-outs, highlighting immune suppression as a key finding, which is not seen in papers analyzing HERV-K. This is consistent with divergent findings when using co-linear HERV-H and HERV-K ISD peptides in macrophage assays [131]. While the immune suppression is described for HERV-H, the specific peptide is highly similar to the ones found in other HERVs of the γ-retrovirus family, including HERV-FRD, but excluding HERV-W, which seems to have inactivating mutations [132]. That HERV-K Env ISD peptides do not act on macrophages in assays where HERV-H is effective could suggest that different mechanisms cause diminished T cell activation following exposure to HERV-K Env transmembrane domain, as observed in other studies using human T cells [110,133]. Such functional studies on immune polarizing properties were not identified in our search of primary cancer studies.

#### 3.14.4. Immunotherapeutic Prospects

A few studies targeting HERVs for cancer immunotherapy have been published and are discussed below.

One principle of HERV-specific immunotherapy is the targeting of HERVs by monoclonal antibodies. This approach was tested successfully in a mouse model, where xenografted HERV-K expressing breast cancer cell lines were inhibited upon the administration of monoclonal anti-HERV-K Env antibodies [95]. As multiple cancers express HERV-K and as multiple HERVs encode envelope proteins (Table 1, Table 2, Table 3, Table 4, Table 5, Table 6, Table 7, Table 8, Table 9, Table 10, Table 11, Table 12 and Table 13), this approach may have broad applicability. 

Similarly, we find that the presence of T cell reactivity toward HERVs has been studied by expanding HERV reactive T cells from cancer patients [83,99]. One study applied CAR T cells engineered to target HERV-K Env and successfully demonstrated HERV-K-specific killing in vitro. Furthermore, CAR T cell treatment significantly reduces tumor burden in melanoma xenografted mice [77]. 

An approach using immunizations against HERVs is known to work in murine models where HERV-K is introduced as a transgene in cancer cell lines [51]. The question becomes, can an immunization approach towards a self-antigen break tolerance? Although there is a large degree of homology between the ERVs in different species, expression and immunogenicity may not be identical. It is therefore difficult to test reliably in vivo. However, much approximated evidence exists from the clinical setting where data are available to indicate that HERVs may indeed be targets of humoral and cellular responses [10,94,99,115]. Of particular interest, cellular graft-versus-tumor occurrence in ccRCC patients receiving hematopoietic stem cell transplant therapy is shown to correlate with recognition of HERV-E T cell epitopes [115,117]. A common concern for these approaches is the risk of adverse effects of targeting a self-antigen. We did identify studies that used healthy adjacent tissue as controls, and these reported that HERVs are rarely found or expressed to a much lower degree in healthy tissues as compared to tumor tissues, exemplified by Krishnamurthy et al. [77]. Sacha et al. reported on a quite extensive tissue library and found that the commercially available anti-HERV K Env antibody HERM-1811-5 does not stain healthy human tissue. Some HERV-K Gag expression was observed with their antibody staining, and this expression was somewhat mirrored in macaques. In spite of this staining, the study showed that the vaccination of macaques against simian ERV-K is immunogenic and safe, underscoring the potential of immunotherapy targeting HERV-K [134]. Notably, only a few studies [77,134] identified in this review included systematic approaches actively looking for HERVs in healthy tissues. More efforts in this regard would be of great value for the field.

With HERVs being immunologically relevant TAAs in cancers [11], an enticing possibility is to combine CPI treatment with a vaccine to stimulate T cells specific for HERVs. Building on data that HERV expression is associated with increased cytolytic activity [135] and response to CPI treatment [10,136], Ficial et al. recently reported an analysis from the CheckMate-025 trial showing that in this large ccRCC cohort, *HERV-E* expression predicts progression-free survival (PFS) in the nivolumab (α-PD-1) arm, but not in the everolimus (mTOR inhibitor) arm [9]. This indicates that CD8+ T cells targeting HERVs may benefit specifically from CPI treatment. Contributing to this notion, a recent study found that HERV-E epitopes, despite their endogenous origin, can be more immunogenic than neoantigens [11], and therefore may pose an ideal target for immunization strategies in combination with CPI. 

Most of these data are based on HERV-E protein expression in ccRCC, where a response is raised naturally [10], but other similar studies investigating other HERVs in other cancer types do exist [137], and since other studies have identified immunogenic HERV epitopes of other origins in cancers [10,67,83,94,99], the potential may be even wider. In this regard, it is important to know that screens for HERV-specific epitopes have been made in a systematic fashion only in hematological malignancies measuring spontaneous responses [83]. Attempts to experimentally validate immunogenic potential are based on painstakingly performing expansion cultures with candidate epitopes and have so far only been reported for HERV-K, -E, and -W [10,94,110,117]. The expansion of this kind of work and its execution in clinical studies of immunotherapy will greatly advance the knowledge on the potential importance of HERVs as adaptive immune therapy targets. Other approaches that have been attempted are based on the elution of MHC-bound peptides and their identification by LC/MS. Such approaches are pursued at least by the biotech company Enara Bio [138], but their results have not been reported in the scientific literature. Whether LC/MS will have a broader utility in HERV proteomics remains to be seen.

Numerous studies were found to investigate demethylation strategies as a type of indirect immunotherapy with the premise that immune responses against tumors may be caused by viral mimicry. The rationale is that HERV expression leads to the occurrence of dsRNA with the deregulation of epigenetic factors [48,49,139]. This association of HERV and dsRNA was reviewed in great detail in 2019, focusing on radiation therapy [140]. Whether the clinical benefit may be affected by the presentation and immune recognition of HERV proteins remains to be established. One study specifically did not find an increase in T cell recognition between pre- and post-treatment with the demethylating compound 5-azacytidine [83].

#### 3.14.5. Antiretrovirals

Antiretrovirals are compounds approved for administration in humans to treat retroviral infections and are as such not developed as cancer therapies. However, as they directly target retroviral protein functions, their applicability may extend into the HERV space. Indeed, antiretroviral therapies have been tested in humans with the aim of targeting HERV-K in 29 patients suffering from amyotrophic lateral sclerosis (ALS), a disease also known to harbor HERV-K expression [141]. Here, antiretroviral compounds abacavir, dolutegravir, and lamivudine were administered over 24 weeks, leading to a progressive decline in HERV-K transcription. This did not translate into a significant decrease in disease progression, but a clear trend was observed towards slower disease progression in patients who also responded to therapy with reduced HERV-K in plasma [141]. 

This approach has also been tested in the field of cancer, though only in murine xenograft models. In a melanoma model, tumor control was achieved using the antiretroviral compound efavirenz [78], and likewise in an ovarian cancer model using demethylating compound romidepsin and antiviral compound vesatolimod [106]. The latter study indicated that this is closely correlated with activation of Toll-like receptor 7 (TLR7) and interferon-γ (IFNγ), suggesting that some degree of viral mimicry function can also be exploited in combination with antiviral therapeutic approaches. Díaz-Carballo et al. previously showed that a wide range of antivirals can sensitize cancer cells to chemotherapies, as shown in the chemotherapy-resistant colorectal HCT8 cell line [50]. 

While clinically promising, the antitumor effects of antiretrovirals such as efavirenz have also been linked to the downregulation of LINE-1 retrotransposon encoded reverse transcriptase [89], which would negatively impact expression. Thus, both increasing and decreasing HERV expression may be associated with clinical responses and the HERV-specific mechanism by which these treatments exert their effects is still unclear.

#### 3.14.6. Diagnostic and Prognostic Use of HERVs

As studies of diagnostic and prognostic value require large cohorts, these studies have typically included transcriptional rather than translational analyses. Thus, transcriptional data should ideally also be reviewed in the context of HERVs as prognostic and diagnostic biomarkers. Wallace et al. found that both HERV-K RNA expression as well as anti HERV-K immunohistochemical staining of tissue correlate with prostate cancer diagnosis [46]. Furthermore, recent findings suggest that transcriptional analysis of HERV expression can predict responsiveness to immunotherapy [142]. Therefore, in this review, the main approach to using HERVs as a diagnostic marker was found to predominantly be measurements of HERV-directed antibodies. This has been shown as a viable strategy with HERV-K and HERV-W in colorectal, prostate, mammary, and dermatological cancers, with some papers correlating this to transcriptional measurements [76,91,96,105]. Notably, increased HERV-K directed antibodies are not only correlated with poor prognosis, but also with the etiology of the cancer; in cancers without a clear etiology defined as sun exposure, the occurrence of antibodies is more frequent and at higher titers [76]. This is consistent with the notion that HERV expression is one option for cancer induction and may not be required if other strong oncogenic drivers such as specific highly potent driver mutations are present [45]. However, in the absence of these drivers, HERV expression may be comparably more important for tumor development. 

### 3.15. Limitations and Bias

In addition to the biases discussed in Section 3.14.1, this study was limited to include only data on translational expression of HERV in cancers, and thus papers with only transcriptional data were omitted. As discussed in Section 3.14.6, using RNA sequencing to quantify HERV expression may be of great diagnostic and/or prognostic value in cancer, but it cannot imply protein expression as dsRNA formation frequently is the result of widespread loss of epigenetic repression [28]. 

When results and interpretations were ambiguous in the included papers, key results were assessed by two different reviewers to identify potential evaluation bias in evaluation of study aims/conclusions. Lastly, only one database was searched systematically. Some papers were added through references in reviews that were identified in the initial search. Of the 72 papers included, 49 were original hits in the initial search. 

## 4. Conclusions

In conclusion, we find that most published studies identify HERV protein expression in a wide range of cancers of differential etiology. This is followed by evidence of humoral and cellular immune responses towards HERVs in cancer patients, indicating that immunological tolerance towards HERVs can be broken. Several therapeutic approaches have been successfully tested in vivo, including monoclonal antibodies, CAR T cells, demethylating compounds and antiviral therapy. Future studies testing these therapies and others in a clinical setting will be of great importance.

## Figures and Tables

**Table 2:** HERV protein expression in neurological cancers.

Indication	HERV	Tissue	Cell Line	Controls	Case/Control	Conclusion	Reference
Glioblastoma	HERV-K	-	U87	-	-	Expression	[43]
Unspecified brain tumor	HERV-K	Serum	-	-	128/0	Seroreactivity	[55]
Schwannoma and meningioma	HERV-K	Tumor	-	Healthy controls	10/10	Expression Tumor function Therapeutic	[69]
Atypical teratoid rhabdoid tumors	HERV-K	Tumor	CHLA 02, CHLA 04, CHLA 05, and CHLA 06	Healthy controls	37/3	Expression Tumor function	[68]

**Table 10:** HERV protein expression in lung cancers.

Indication	HERV	Tissue	Cell Line	Controls	Case/Control	Conclusion	Reference
NSCLC	HERV-K	-	A549	-	-	Expression	[43]
SCLC	HERV-K	-	H69	-	-	Expression Tumor function Therapeutic	[78]

NSCLC: Non-small cell lung carcinoma. SCLC: Small cell lung carcinoma.

**Table 11:** HERV protein expression in pancreatic cancers.

Indication	HERV	Tissue	Cell Line	Controls	Case/Control	Conclusion	Reference
Pancreatic adenocarcinoma	HERV-H	-	Panc-1	-	-	Expression Tumor function	[81]
Pancreatic adenocarcinoma	HERV-K	Serum Tumor	Panc-1, Panc-2, HPDE-E6E7	Adjacent Healthy controls	106/40 1/1	Expression Tumor function Seroreactivity	[101]
Pancreatic adenocarcinoma	HERV-K	-	Panc-1	-	-	Expression Tumor function	[43]

**Table 12:** HERV protein expression in endocrine cancers.

Indication	HERV	Tissue	Cell Line	Controls	Case/Control	Conclusion	Reference
Pituitary adenoma	HERV-W	Tumor	-	From sellar exploration where no adenoma was found	117/15	Expression Tumor function	[120]

**Table 13:** HERV protein expression in sarcomas.

Indication	HERV	Tissue	Cell Line	Controls	Case/Control	Conclusion	Reference
Kaposi’s sarcoma	HERV-K	Blood	BCBL-1, BC-1, BC-3 BCP-1	Blood from KSHV-neg HIV patients	11/10	Expression Tumor function	[121]

KSHV: Kaposi’s sarcoma associated herpes virus.

## Data Availability

All data in this paper were acquired through database search in the PubMed/Medline database using Medical Search Headings (MeSH), and from references in identified reviews. A minimal number of papers were added as a result of additional searches when designing the discussion. A list of papers included in the analysis is available upon request.
